# Statistical Accuracy of Administratively Recorded Race/Ethnicity in the Military Health System and Race/Ethnicity Ascertained via Questionnaire

**DOI:** 10.1007/s40615-025-02351-7

**Published:** 2025-03-21

**Authors:** Jordan McAdam, Stephanie A. Richard, Cara H. Olsen, Celia Byrne, Shawn Clausen, Amber Michel, Brian K. Agan, Robert O’Connell, Timothy H. Burgess, David R. Tribble, Simon Pollett, James D. Mancuso, Jennifer A. Rusiecki

**Affiliations:** 1 Department of Medicine, Uniformed Services University of the Health Sciences, Bethesda, MD USA; 2 Henry M. Jackson Foundation for the Advancement of Military Medicine, Inc, Bethesda, MD USA; 3 Department of Preventive Medicine and Biostatistics, Uniformed Services University of the Health Sciences, Bethesda, MD USA; 4 Infectious Diseases Clinical Research Program, Department of Preventive Medicine and Biostatistics, Uniformed Services University of the Health Sciences, Bethesda, MD USA; 5 Epidemiology and Analysis Section, Defense Health Agency, Armed Forces Health Surveillance Division, Silver Spring, MD USA

**Keywords:** Health inequities, Military health, Military health services, Electronic health records

## Abstract

**Background:**

Unequal disease burdens such as SARS-CoV-2 infection rates and COVID-19 outcomes across race/ethnicity groups have been reported. Misclassification of and missing race and ethnicity (race/ethnicity) data hinder efforts to identify and address health disparities in the US Military Health System (MHS); therefore, we evaluated the statistical accuracy of administratively recorded race/ethnicity data in the MHS Data Repository (MDR) through comparison to self-reported race/ethnicity collected via questionnaire in the Epidemiology, Immunology, and Clinical Characteristics of Emerging Infectious Diseases with Pandemic Potential (EPICC) cohort study.

**Methods:**

The study population included 6009 active duty/retired military (AD/R) and dependent beneficiaries (DB). Considering EPICC study responses the “gold standard,” we calculated sensitivity and positive predictive value (PPV) by race/ethnicity category (non-Hispanic (NH) White, NH Black, Hispanic, NH Asian/Pacific Islander (A/PI), NH American Indian/Alaskan Native (AI/AN), NH Other, missing/unknown).

**Results:**

Among AD/R, the highest sensitivity and PPV values were for NH White (0.93, 0.96), NH Black (0.90, 0.92), Hispanic (0.80, 0.93), and NH A/PI (0.84, 0.95) and lowest for NH AI/AN (0.62, 0.57) and NH Other (0.09, 0.03). The MDR was missing race/ethnicity data for approximately 63% of DB and sensitivity values, though not PPV, were comparatively much lower: NH White (0.35, 0.88), NH Black (0.55, 0.89), Hispanic (0.13, 1.00), and NH A/PI (0.28, 0.84).

**Conclusions:**

Our evaluation of MDR race/ethnicity data revealed misclassification, particularly among some minority groups, and substantial missingness among DB. The potential bias introduced impacts the ability to address health disparities and conduct health research in the MHS, including studies of COVID-19, and needs further examination.

**Supplementary Information:**

The online version contains supplementary material available at 10.1007/s40615-025-02351-7.

## Introduction

Race and ethnicity (race/ethnicity) are complex constructs that influence identity and social relations [1]. Race/ethnicity identity and classification systems can vary geographically, temporally, and contextually [1]. In the USA, racial and ethnic disparities in health and healthcare have been widely reported, including during the COVID-19 pandemic [2–5]. Disparities in health and healthcare occur when subsets of a population experience worse health determinants, outcomes, or access to healthcare than others [2].

Universal healthcare systems, such as the Military Health System (MHS), that are designed for equal access and are geographically distributed have been suggested to minimize disparities in access to healthcare. Research on racial and ethnic disparities in health outcomes and healthcare utilization within the MHS have had mixed findings. Studies have reported racial and/or ethnic disparities in medical deployability [6]; mental health treatment [7, 8]; acute low back pain [9]; cancer screening, treatment, and survival [10–13]; maternal and neonatal health outcomes [14, 15]; and chronic disease prevalence and outcomes [16–18] within the MHS, while other studies have not found evidence of racial and/or ethnic disparities related to patient satisfaction [19], physical and mental health status [19], mortality [20], readmission outcomes [20], and cancer treatment and survival [21–23]. The prevalence of disparities in health outcomes and healthcare utilization (including during COVID-19) as well as the factors driving those disparities are still understudied within the MHS. Elimination of health disparities and achievement of health equity are core aims within the MHS and nationwide [24, 25].

**Fig. 1:**
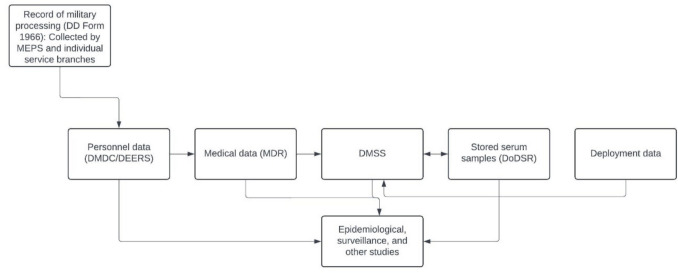
Relationships between the Defense Manpower Data Center (DMDC)/Defense Enrollment Eligibility Reporting System (DEERS), Military Health System Data Repository (MDR), Department of Defense Serum Repository (DoDSR), and Defense Medical Surveillance System (DMSS)

There are many sources of race/ethnicity data within the Military Health System Data Repository (MDR) (Supplemental Table 1), and their collection processes vary. Although written documentation is limited, self-reported race/ethnicity data are obtained at both Military Entrance Processing Stations (MEPS) and by individual service branches for service members upon entry into the military as part of their administrative record; data are also collected during healthcare encounters. For MHS dependent beneficiaries, there is not a race/ethnicity field on enrollment form needed for the Uniformed Services health care program, TRICARE [26]. For race/ethnicity data collected at MEPS and by the Service Branches, data are transmitted to and standardized by the Defense Enrollment Eligibility Reporting System (DEERS), a large database managed by the Defense Manpower Data Center (DMDC) (A. Ricci, personal communication, March 16, 2024). Race/ethnicity data from DEERS are imported into the MDR, a medical health encounter data repository maintained by the military containing information regarding inpatient and outpatient health encounters received from both military treatment facilities (MTFs) and civilian facilities, for which care is billed to the military. Data originally derived from DEERS also feeds into the Defense Medical Surveillance System (DMSS) (which recodes race/ethnicity data [S. Stahlman, personal communication, March 11, 2024]), which links with the Department of Defense Serum Repository (DoDSR), an archive of sera drawn from servicemembers for medical surveillance [27, 28]. The MDR, DMSS, and DoDSR are valuable resources that offer the opportunity for comprehensive public health surveillance [28] and are utilized in population health and clinical research. The relationships between these data sources and resources are outlined in Fig. 1.

When studying health inequities within a system or population, misclassification may occur if race/ethnicity are not measured accurately [1]. Self-identification is considered the gold standard for race/ethnicity data collection and is the preferred approach for race/ethnicity ascertainment according to the Office of Management and Budget (OMB) 1997, which provides minimum standards for collecting data race/ethnicity for Federal reporting, and the Department of Health and Human Services 2011 standards [29, 30]. Additionally, many studies have used self-reported race/ethnicity as the gold standard. Missing race/ethnicity data may also pose a problem. Two common approaches to address missing data, excluding subjects with incomplete data for variables of interest from analyses (i.e., listwise deletion or complete-case analyses) and imputation of missing values, may introduce bias [31]. Understanding the accuracy of the MDR with regard to race/ethnicity data is relevant due to the potential uses of MDR and DMSS data, as well as DoDSR specimens to advance population and clinical health research as well as address health equity issues, including research related to infectious diseases, such as COVID-19. Additionally, understanding the accuracy of administrative race/ethnicity records is critical as the racial and ethnic composition of the US military changes over time [32]. Past studies have evaluated the agreement between and accuracy of sources of race/ethnicity data among or within Medicare and Medicaid beneficiaries [33–36], patients served by community health centers or other medical centers [37–40], cancer survivors [41], cancer registries [42, 43], veterans and the Department of Veterans Affairs (VA) [44–49], and other databases and repositories [50–52]. A recent systematic review evaluating the accuracy of race/ethnicity data in US-based healthcare databases found that Asian, American Indian and Alaskan Native (AI/AN), and Pacific Islanders were the most misclassified across databases [29].

Ascertainment of race/ethnicity among service members and dependent beneficiaries (hereafter collectively referred to as MHS beneficiaries) is critical for many areas of health research: identifying and monitoring health disparities [30, 53], improving public health interventions to eliminate health disparities [54], improving quality of care and outcomes for patients, and identifying high-risk populations for specific outcomes [55]. To date, we are not aware of any studies to evaluate the accuracy of MDR-recorded race/ethnicity data, which are the basis for much of the retrospective MHS health research [26]. Because this data source is often used in health studies to assign the race/ethnicity of MHS beneficiaries and the MHS beneficiary population continues to become more racially and ethnically diverse [32], it is important to evaluate the quality of these data. Therefore, we evaluated the statistical accuracy of administratively recorded race/ethnicity data within the MDR through comparison to the gold standard, race/ethnicity data collected via questionnaire in the Epidemiology, Immunology, and Clinical Characteristics of Emerging Infectious Diseases with Pandemic Potential (EPICC) cohort study, among MHS beneficiaries.

## Methods

### Population

The EPICC observational cohort study, described previously [56, 57], included US military active duty service members, retirees, and their family members (of any age) that were enrolled at 10 MTFs between March 2020 and May 2022 to explore risk factors for and characteristics of SARS-CoV-2 infections. MTF-based enrollment was augmented by an online recruitment pathway. Eligible participants met one or more of the following criteria: (1) presented a positive SARS-CoV-2 test; (2) met criteria for testing for SARS-CoV-2 (including presenting with a COVID-19-like illness or had a high-risk SARS-CoV-2 exposure). In 2021, an additional eligibility criterion was added to include (3) those who received a licensed COVID-19 vaccine. Demographic data, including self-reported race/ethnicity, were collected at enrollment through participant questionnaires. Questionnaire data was augmented by case report forms and abstraction of MDR data as part of consent. The EPICC study population consists of 7911 MHS beneficiaries; however, 1857 participants who were enrolled at an MTF without online participant-reported enrollment surveys and 45 participants not classified as active duty, retired military, or dependent beneficiary (i.e., another military status or missing information) were excluded from our analyses, for a final population of 6009 (Supplemental Fig. 1).

### Race/Ethnicity Ascertainment

#### MDR VM6BEN Race/Ethnicity Data

To the best of our knowledge, upon entrance into the military and at MEPS, each branch of service (e.g., Army, Air Force, Navy, Marines, and Coast Guard) collected race/ethnicity information via the service member completing DD Form 1966 (https://www.esd.whs.mil/Portals/54/Documents/DD/forms/dd/dd1966.pdf, last accessed May 1, 2024) since at least 1975 [58], but racial and ethnic categories included on DD Form 1966 have changed over time. The categories available at the time of our data collection included: (1) AI/AN; (2) Asian; (3) Black or African American; (4) Native Hawaiian or Other Pacific Islander (NH/OPI); and (5) White, and service members could select all that apply. Ethnicity categories included on DD Form 1966 were (1) Hispanic or Latino and (2) Not Hispanic or Latino (NH). DD Form 1966 changed again as of June 2024 and now includes (1) AI/AN; (2) Asian; (3) Black or African American; (4) Hispanic or Latino; (5) Middle Eastern or North African (ME/NA); (6) NH/OPI; (7) White; and (8) Other. The DD Form 1966 data are shared with DEERS as part of personnel service records, and the DMDC standardizes and recodes the race/ethnicity data (A. Ricci, personal communication, March 16, 2024), according to Department of Defense Instruction (DoDI) 1336.05 (Active Component) [59] and DoDI 7730.54 (Reserve Component) [60]. The DEERS database contains information for each uniformed service member (active duty, retirees, or member of a Reserve Component) [61].

Sponsors (i.e., service members) must register their dependent beneficiaries in DEERS [61]. The current application for DEERS enrollment of a dependent beneficiary, DD Form 1172 (https://www.cac.mil/portals/53/documents/dd1172-2.pdf, last accessed June 24, 2024), does not ascertain race/ethnicity information for dependent beneficiaries [26]. Despite this, dependent beneficiary race/ethnicity information is available in DEERS for some, which could be due to prior military service, civilian or contractor jobs within the military, or other factors (A. Ricci, personal communication, March 16, 2024). Due to these differences in race/ethnicity ascertainment, we evaluate the accuracy of MDR race/ethnicity information separately for service members and dependent beneficiaries in our analyses.

There are several sources of race/ethnicity data within the MDR available for researchers, which are outlined in Supplemental Table 1 and Supplemental Table 2. The EPICC study obtained race/ethnicity data from the MDR *VM6* database in the MDR (Supplemental Table 1): “RACE_CD” and “RACE_ETHNC_CD.” These variables were selected as these are commonly employed in MHS population health services research (A. Banaag, personal communication, March 22, 2024). For RACE_CD, the options for race categories are (1) White, (2) Black, (3) Asian or Pacific Islander (A/PI), (4) AI/AN, (5) Other, and (6) Unknown. For RACE_ETHNC_CD, the options for race/ethnicity categories are (1) White, NH; (2) Black, NH; (3) Hispanic; (4) Asian or Pacific Islander; (5) AI/AN; (6) Other; and (7) Unknown. RACE_CD was used to evaluate MDR race within this analysis, while RACE_ETHNC_CD was used to evaluate MDR ethnicity. “Unknown,” until 2023, was a code that could be entered into a field, while “missing” implies the data is missing or unavailable (A. Ricci, personal communication, March 16, 2024). A single race/ethnicity is reported by the MDR variables RACE_CD and RACE_ETHNC_CD. Hereafter, we specify NH White, NH Black, NH A/PI, NH AI/AN, and NH Other in analyses, where we include Hispanic or Latino ethnicity.

#### EPICC Questionnaire Race/Ethnicity Data

Self-reported race/ethnicity information used in this analysis was collected upon enrollment via a self-administered online questionnaire that was introduced in the EPICC study in October 2020. Participants could select as many race categories as applicable from the following: (1) White; (2) Black; (3) Asian; (4) AI/AN; (5) NH/OPI; and (6) Other. For those who selected “Other,” participants could write in their race identification. For ethnicity, participants could select from the following categories: (1) Hispanic or Latino or (2) NH. Participants could also select “Prefer not to answer” for both race (2.51% reported) and ethnicity (5.13% reported) questions, in which case they were excluded from our analyses. For the purposes of comparison with the MDR RACE_CD and RACE_ETHNC_CD categories, we combined the Asian and NH/OPI race categories into an A/PI category.

### Statistical Analyses

The accuracy of the MDR RACE_CD and RACE_ETHNC_CD variables compared to the EPICC questionnaire gold standard [62, 63] was assessed by calculating sensitivity, positive predictive value (PPV), and Cohen’s kappa coefficient for each race/ethnicity category using *n* × *n* tables. We also calculated simple kappa coefficients to calculate overall agreement between the MDR RACE_CD and RACE_ETHNC_CD variables with the EPICC questionnaire data. Although kappa statistics are traditionally used to evaluate interrater agreement and reliability, designating one measure as the reference group (in our case, EPICC questionnaire data) does not violate required assumptions for the use of the kappa [64]. The metrics utilized in the study are described in greater detail in Supplemental Table 3.

Initially, to evaluate how participants reporting multiple races in the EPICC questionnaire were recorded in the MDR RACE_CD variable, which reports a single race, we created an *n* × *n* matrix comparing race category (MDR RACE_CD) and combination of race categories (EPICC questionnaire) recorded by each source. Similarly, we tabulated the counts and percents of MDR RACE_CD-classified race for participants in EPICC who indicated NH/OPI race on the questionnaire to give further insight into agreement within this population. Due to small counts among dependent beneficiaries, we included only active duty and retired military (hereafter referred to as “active duty/retired”) in the analyses of participants reporting multiple races in the EPICC questionnaire and when focusing on EPICC NH/OPI participants.

We evaluated statistical accuracy of the MDR data and agreement between the MDR (MDR RACE_ETHNC_CD and RACE_CD) data and EPICC questionnaire data using two approaches:

1. Evaluating race/ethnicity (*NH White*, *NH Black*, *Hispanic/Latino (any race)*, *NH A/PI*, *NH AI/AN*, *NH Other*)
2. Evaluating race only (*White*, *Black*, *A/PI*, *AI/AN*, *Other*)

We excluded participants with “Prefer not to answer” for race/ethnicity and race in the EPICC questionnaire, respectively. We excluded participants who reported multiple races in the EPICC questionnaire, as the RACE_ETHNC_CD and MDR RACE_CD variables report a single race. Both approaches were evaluated (1) including a category for missing/unknown race/ethnicity information from the MDR RACE_ETHNC_CD or RACE_CD variables and/or EPICC questionnaire data and (2) excluding participants with missing/unknown race/ethnicity information from the MDR RACE_ETHNC_CD or RACE_CD variables and/or EPICC questionnaire data.

Active duty/retired participants and dependent beneficiaries were evaluated in our analyses separately. We stratified by age group (< 18, 18–44, 45–64, and ≥ 65 years) and sex (male, female) to evaluate differences. We could not evaluate year of entry into the military within our study population due to limitations of the MHS data linked to EPICC; therefore, we consider age group a proxy of era of entry into the military. Additionally, among active duty/retired, we stratified by branch of service. All analyses were performed using SAS, version 9.4 (SAS Institute, Inc, Cary, NC).

## Results

The characteristics of active duty, retired military, and dependent beneficiary EPICC participants (*n* = 6009) are outlined in Table 1. The majority of participants were 18–44 years old (80.2%), male (64.7%), and active duty (80.7%). Over 40% of service members were in the Army, while less than 10% were in the Marines or Coast Guard. A descriptive summary of recorded race/ethnicity, by data source (MDR RACE_CD/RACE_ETHNC_CD or EPICC questionnaire) and service (active duty/retired or dependent beneficiary), is presented in Table 2. Race/ethnicity missingness was higher for the MDR race (RACE_CD) (1.6%) variable than for the EPICC race (0.3%) questionnaire data but lower for the MDR ethnicity (RACE_ETHNC_CD) (1.6%) variable than for the EPICC ethnicity (8.8%) questionnaire data among the active duty/retired group. Most dependent beneficiaries were missing race/ethnicity information in the MDR RACE_CD (63.9%) and RACE_ETHNC_CD (64.1%) variables.

**Table 1:** Characteristics of active duty, retired military, and dependent beneficiary EPICC participants, *n* = 6009

Characteristic	*N* (%)
Age (years)	
< 18	3 (0.1)
18–44	4820 (80.2)
45–64	1034 (17.2)
≥ 65	152 (2.5)
Sex	
Male	3887 (64.7)
Female	2120 (35.3)
Other	2 (< 1.0%)
Service	
Active duty	4851 (80.7)
Dependent beneficiary	715 (11.9)
Retired military	443 (7.4)
Branch of service^a^	
Air Force	1186 (22.5)
Army	2264 (42.9)
Marines or Coast Guard	490 (9.3)
Navy	1242 (23.5)
Other	95 (1.8)
Missing	17

^a^Branch of service recorded for dependents (i.e., the branch of service for the dependent’s sponsor) not presented

**Table 2:** Descriptive race/ethnicity information by data source and service

***Active duty + retired military*** **(*n* = 5294)**		
**Race**	**EPICC Questionnaire**^a^	**MDR**^b^
	***N* (%)**	***N* (%)**
White	3689 (69.9)	3794 (72.9)
Black	504 (9.6)	572 (11.0)
Asian/Pacific Islander^c^	293 (5.6)	357 (6.9)
American Indian/Alaskan Native	41 (0.8)	53 (1.0)
Other	208 (3.9)	384 (7.4)
Multiple	400 (7.6)	–
Unknown	–	48 (0.9)
Prefer not to answer	141 (2.7)	–
Missing	18	86
***Ethnicity***	***N (%)***	***N (%)***
Non-Hispanic or Latino	3659 (75.8)	4393 (84.8)
Hispanic or Latino	885 (18.3)	786 (15.2)
Prefer not to answer	283 (5.9)	–
Missing	467	86
***Dependent beneficiaries*** (***n* = 715)**		
**Race**	**EPICC Questionnaire**^**a**^	**MDR**^**b**^
	***N* (%)**	***N* (%)**
White	515 (72.5)	186 (26.7)
Black	46 (6.5)	27 (3.9)
Asian/Pacific Islander^c^	61 (8.6)	23 (3.3)
American Indian/Alaskan Native	4 (0.6)	3 (0.4)
Other	30 (4.2)	19 (2.7)
Multiple	44 (6.2)	–
Unknown	–	439 (63.0)
Prefer not to answer	10 (1.4)	–
Missing	5	18
***Ethnicity***	***N* (%)**	***N* (%)**
Non-Hispanic or Latino	490 (75.7)	240 (93.4)
Hispanic or Latino	132 (20.4)	17 (6.6)
Prefer not to answer	25 (3.9)	–
Missing	68	458

*EPICC* The Epidemiology, Immunology, and Clinical Characteristics of Emerging Infectious Diseases with Pandemic Potential study, *MDR* Military Health System Data Repository

^a^Responses to EPICC study questionnaire

^b^RACE_CD and RACE_ETHN_CD variables within the MDR

^c^EPICC included separate race categories for Asian and for Native Hawaiian or Other Pacific Islander, while the MDR reports Asian/Pacific Islander as a single race. We have combined the EPICC-ascertained Asian and Native Hawaiian or Other Pacific Islander categories into Asian/Pacific Islander in order to align with the MDR classifications

There were not consistent trends comparing active duty/retired identifying as multiple races in the EPICC questionnaire and their race recorded in the MDR RACE_CD variable. The majority of participants indicating multiple races identified as White as well as Asian, Black, AI/AN, or NH/OPI in the EPICC questionnaire. Patterns of race recorded in the MDR RACE_CD variable were not clear or consistent for those responding as White and Asian [MDR race: A/PI (38.5%); Black (3.4%); Other (29.9%); Unknown (5.1%); and White (23.1%)], White and Black [MDR race: AI/AN (1.6%); A/PI (3.2%); Black (41.1%); Other (32.3%); Unknown (6.5%); and White (15.3%)], White and AI/AN [MDR race: AI/AN (12.2%); A/PI (4.4%); Black (5.2%); Other (20.0%); Unknown (7.0%); and White (51.3%)], and White and NH/OPI [MDR race: A/PI (31.6%); Other (31.6%); Unknown (2.6%); and White (34.2%)] (results not shown). Of the 119 EPICC NH/OPI participants, 50.9% were classified in the MDR RACE_CD as A/PI, 22.9% classified as Other, and 15.3% as White (results not shown). Over half of the NH/OPI participants in the EPICC questionnaire reported multiple races. Excluding NH/OPI persons reporting multiple races in the EPICC questionnaire, 31 of the 46 NH/OPI participants were classified as A/PI in the MDR RACE_CD (results not shown).

Table 3 presents counts, column percent (equivalent to sensitivity for concordant categories only), and row percent (equivalent to PPV for concordant categories only) comparing MDR RACE_ETHNC_CD and EPICC questionnaire race/ethnicity data, including participants missing race/ethnicity information in the MDR RACE_ETHNC_CD and/or the EPICC questionnaire. For active duty/retired, the highest sensitivity and PPV were found for NH White race (0.93, 0.96), while the lowest were for NH Other race (0.09, 0.03). The simple kappa statistic summarizing agreement between the MDR RACE_ETHNC_CD and EPICC questionnaire for active duty/retired was 0.80. For dependent beneficiaries, the highest sensitivity was for NH Black race (0.55) and the highest PPV was for Hispanic ethnicity (1.00), though this was based on small counts. The lowest sensitivity was for Hispanic or Latino ethnicity (any race) (0.13) and the lowest PPV was for NH A/PI race (0.84). The simple kappa statistic summarizing agreement between the MDR RACE_ETHNC_CD and EPICC questionnaire for dependent beneficiaries was 0.16. Supplemental Table 4 presents this information excluding participants missing MDR RACE_ETHNC_CD and/or EPICC questionnaire race/ethnicity information. Sensitivity and PPV values were higher when excluding participants missing race/ethnicity information for both active duty/retired and dependent beneficiaries, and simple kappa coefficients rose (active duty/retired 0.83; dependent beneficiaries 0.71).

**Table 3:**
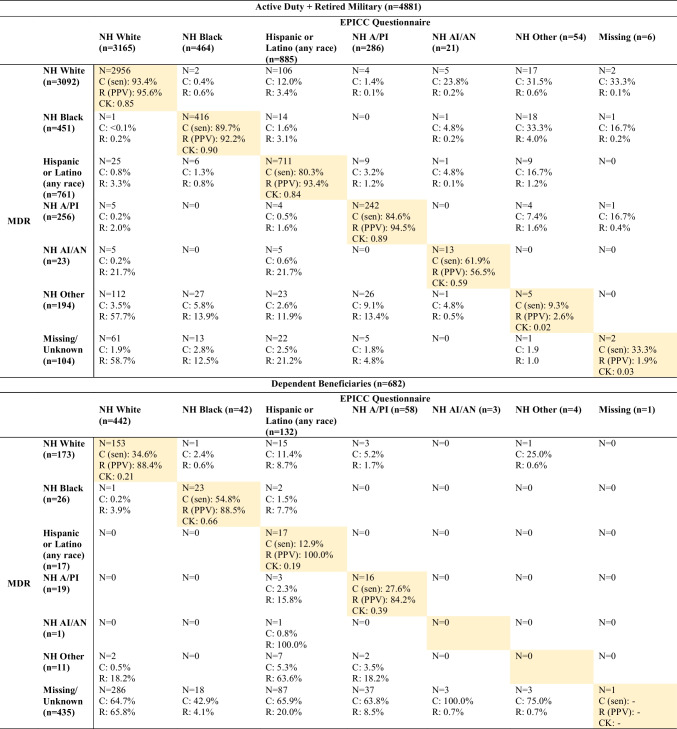
Counts, sensitivity, PPV, and Cohen’s kappa coefficients by race/ethnicity category comparing MDR and EPICC questionnaire data, including missing/unknown data

Shading indicates matched race/ethnicity categories. Row percent (R) is equivalent to positive predictive value and column percent (C) is equivalent to sensitivity. Participants indicating “Prefer not to answer” for race in EPICC are excluded from these analyses. “MDR” refers to the MDR’s RACE_ETHNC_CD variable

*AI/AN* American Indian/Alaskan Native, *A/PI* Asian/Pacific Islander, *C* column percent, *CK* Cohen’s kappa coefficient, *EPICC* The Epidemiology, Immunology, and Clinical Characteristics of Emerging Infectious Diseases with Pandemic Potential study, *MDR* Military Health System Data Repository, *PPV* positive predictive value, *R* row percent, *sen* sensitivity

Findings for race-only analyses were similar to the race/ethnicity analyses. Supplemental Tables 5 and 6 present counts, column percent, row percent, and Cohen’s kappa coefficients comparing race only between the MDR RACE_CD and EPICC questionnaire. Supplemental Table 5 includes participants missing race information in the MDR RACE_CD and/or EPICC questionnaire, while Supplemental Table 6 excludes participants missing race information in the MDR RACE_CD and/or EPICC questionnaire.

Stratification by age group, sex, and branch of service did not reveal any meaningful differences.

## Discussion

To our knowledge, this is the first study to evaluate concordance between MDR RACE_CD and RACE_ETHNC_CD variables and questionnaire-reported race/ethnicity among MHS beneficiaries. In this population, there was high missingness of MDR race/ethnicity data for dependent beneficiaries (> 60%) but comparatively low missingness for active duty/retired participants (< 5%). The accuracy of MDR was moderate to strong when compared to the EPICC study reporting of race/ethnicity for NH White, NH Black, Hispanic or Latino, and NH A/PI categories but was relatively low among NH AI/AN and NH Other race categories within the active duty/retired group. The MDR reporting of race/ethnicity data for dependent beneficiaries was poor for all race/ethnicity categories. When excluding missing data, statistical accuracy measures for all race/ethnicity categories among both active duty/retired and dependent beneficiaries rose.

Our findings are similar to previous studies outside the MHS. Two studies, one using birth certificate race/ethnicity data and the other using self-reported race/ethnicity as the gold standard, evaluated the quality of hospital discharge and cancer registry race and ethnicity data, respectively. The studies found NH White and NH Black race categories had relatively high sensitivity and PPV, while Hispanic ethnicity and NH Asian race had moderate sensitivity and high PPV [40, 42]. Similar findings were found when comparing self-reported race/ethnicity data to administratively recorded VA [47] and Medicare [36] data. Other studies, however, have found much lower agreement for Hispanic ethnicity compared to our findings. Comparing self-reported race/ethnicity compared to that recorded in a health maintenance organization, one study found sensitivities and PPVs were high for NH Black (0.95 and 0.95, respectively) and NH White (0.98 and 0.94, respectively) race categories, lower for the NH Asian race category (0.88 and 0.95, respectively), and lowest in the Hispanic (0.55 and 0.81, respectively) and NH AI/AN (0.47 and 0.50, respectively) race/ethnicity categories [38]. A study of race/ethnicity agreement between Medicare and Medicaid administrative databases found the relative agreement rate was 84% for NH White, 74% for NH Black, 61% for NH Other, 23% for Hispanic, and only 5% for NH Asian race/ethnicity categories when using Medicare data as the reference [34]. Together, these studies, with ours, suggest NH White and NH Black race/ethnicity categories are most likely to be accurately and consistently classified in administrative databases, while Hispanic, NH Asian, and AI/AN individuals are often misclassified.

Our findings indicated a high number of dependent beneficiaries classified as “Unknown” or with missing race/ethnicity data in the MDR. Reporting has suggested race/ethnicity data are mostly available for service members and retirees, but missing for dependent beneficiaries [26], as corroborated by our findings. Potential factors that may influence the missingness of race/ethnicity data for dependent beneficiaries have been suggested: (1) DEERS may copy race/ethnicity of sponsors to blank dependent beneficiary records; (2) data entry personnel do not always confirm race/ethnicity of dependent beneficiaries at DEERS enrollment; (3) the application for a military identification card and/or DEERS enrollment does not include a field for race/ ethnicity to ascertain the information from dependent beneficiaries; and (4) servicemembers and beneficiaries are unable to view or edit race/ ethnicity in their DEERS record [26]. The MDR data dictionary notes for the MDR RACE_CD and RACE_ETHNC_CD variables included in our study “This variable is unreliable for non-sponsors. Users should not assume that the race/ethnicity of a sponsor is the same as the race of a family member” (https://www.health.mil/Reference-Center/Technical-Documents/2024/05/31/MDR-Data-Dictionary, last accessed June 24, 2024). When excluding missing dependent beneficiary data, the MDR race/ethnicity data had greater accuracy, though this was based on relatively small counts. Given the high percent missingness of race/ethnicity data in the MDR for dependent beneficiaries, both listwise deletion analyses and imputation are not feasible approaches to address the missing data problem, which could limit the ability to evaluate health disparities within this population and warrants attention.

We found particularly low accuracy of MDR race/ethnicity for AI/AN compared to other race/ethnicity categories included in our analyses, with the potential for race misclassification in the MDR of over half of AI/AN EPICC questionnaire respondents. These findings are similar to studies in other populations [29, 33, 36, 38, 39, 41, 42, 44, 45, 47, 50, 63]. The racial misclassification of AI/AN individuals across many databases is a complex issue. It has been suggested that factors at the systems, policy, and individual levels may contribute to this occurrence [65] and reporting self-identification of AI/AN race has changed over time [66]. Although we stratified by age as a proxy for era of entry into the military, we did not identify trends related to AI/AN agreement over time, though there were small counts of AI/AN EPICC participants. Additionally, it has been reported that only 40% of AI/AN in the US identify as one race alone [67]; therefore, AI/AN race may not be accurately captured by the MDR as it reports a single race. Future studies may consider oversampling AI/AN beneficiaries to better represent this population in MHS-related research, given the documented health disparities for health outcomes including COVID-19 [68], and consider adjusting for racial miscoding.

High misclassification of the “Other” race category in the MDR RACE_CD was found in our study. Although not all participants identifying as “Other” race provided a write-in response in the EPICC questionnaire, over half (128/193) self-identified as Hispanic or Latino or likely Hispanic or Latino identities: Central American or Mexican, Hispanic, Latino/a or Latin American, or Puerto Rican (Supplemental Table 7). Similar findings have been noted in other studies. One study showed that two-thirds of mothers of Hispanic ethnicity reported their race as “some other race” [69], and a qualitative study found that Hispanic/Latino patients reported problems in not being able to document “Hispanic/Latino” as their race [70]. Similarly, in a study of statewide hospital discharge data, there was a significantly higher rate of missing race data among Hispanic/Latino patients compared to other patients [71]. The OMB currently defines “Hispanic or Latino” as US residents of Cuban, Mexican, Puerto Rican, South or Central America, or “other” Spanish cultures or origins [72]. According to a Pew Research Center survey, two-thirds of Latinos say their Hispanic background is part of their racial identity, despite the US Census Bureau considering Hispanic or Latino as an ethnicity [73]. The complexities surrounding Hispanic or Latino identity have led to recommendations such as using combined race/ethnicity questions and open-ended race/ethnicity questions [74], or providing specific definitions for Hispanic and/or Latino [75]. Following the guidance for the new minimum race/ethnicity reporting categories (SPD 15) from the OMB could address this issue. The new OMB standards include the following race/ethnicity categories—AI/AN, Asian, Black or African American, Hispanic or Latino, Middle Eastern or North African, NH/OPI, and White—and race/ethnicity, as currently defined by the OMB, will not be presented separately [76]. DD Form 1966 has been recently updated with these new OMB standards (https://www.esd.whs.mil/Portals/54/Documents/DD/forms/dd/dd1966.pdf, last accessed May 29, 2024), though it is unknown if or when these updates will be reflected in MDR race/ethnicity variables.

Although DD Form 1966 includes “Asian” and “Native Hawaiian or Other Pacific Islander” (i.e., the OMB standard categories [disaggregated race categories]), the MDR RACE_ETHNC_CD and RACE_CD variables aggregate these categories into a single A/PI category for researchers, which does not comply with OMB standards. Additionally, studies have indicated that 30% of individuals that identify as multiracial are Asian American or NH/OPI [77]. NH/OPI individuals often have worse health outcomes than those who are Asian and White, such as obesity and diabetes/pre-diabetes prevalence [78]. One study found that Native Hawaiians were significantly less likely than non-native Hawaiians to be correctly classified in administrative hospital records, and the number of COVID-19-related hospitalizations was approximately 8% higher for Native Hawaiians and 4% higher for Pacific Islanders when using self-identified race/ethnicity data compared to hospital data [79]. Additionally, studies have suggested that aggregating A/PI masks a wide variation in access to health services and in health outcomes by ethnic subgroup [80, 81]. Together, these results support other recommendations, including from the Health Systems Subcommittee within the Defense Health Board, an independent Federal advisory committee, to (1) disaggregate Asian and NH/OPI categories in the MDR and (2) include a “multi-race” category in the MDR [26].

Changes in racial or ethnic identify can occur at both population and individual levels over time [82]; however, self-reported race/ethnicity may also change based on the format of the question, the format of ascertainment (e.g., oral reporting vs online questionnaire), or other factors. Census categories and descriptions of race/ethnicity have changed each decade [83], and though there are limited records on how race/ethnicity have been ascertained in the Armed Forces historically, it has also varied over time. A Pew Research Center poll found that more than 10 million Americans checked different race or Hispanic-origin boxes in the 2010 census compared to the 2000 census, stating that “Hispanics, Americans of mixed race, American Indians and Pacific Islanders were among those most likely to check different boxes from one census to the next” [84]. Additionally, a study of Census Bureau data from the 2000 and 2010 censuses found that race response change was relatively common among persons who reported AI/AN or NH/OPI in a multiple-race response group [85]. Further study into racial and ethnic misclassification in the MDR by era of entry into the military is warranted. The DEERS website does not provide information regarding updating race/ethnicity with Service personnel offices, as they do for name, sex, and other fields [26], which may not allow for personnel records to be changed to accurately reflect an individual’s understanding or identification of their race/ethnicity over time. Establishing mechanisms to allow all MHS beneficiaries to view and update their race or ethnicity in DEERS may address this issue.

### Implications and Future Directions

#### SARS-CoV-2 and COVID-19

Reports have highlighted unequal burdens of disease across race/ethnicity groups, such as SARS-CoV-2 infection rates and COVID-19 outcomes, but the magnitude of disparities has been uncertain in some cases due to missing race/ethnicity information in surveillance data [86]. Complete cases analyses can bias race/ethnicity disparity estimates if race/ethnicity data are not missing at random or missing completely at random [87]. One study found that accounting for missing race/ethnicity information showed greater differences in SARS-CoV-2 infection rates comparing most minority race/ethnicity categories to White race, and the authors concluded that conducting only complete case/listwise deletion analyses or using imputation, without bias-adjustment, could underestimate the magnitude of disparities in COVID-19 morbidity and mortality [86, 88]. These results, in addition to our findings, highlight the importance of considering missing race/ethnicity data as well as misclassified race/ethnicity information when evaluating disparities in disease burden and health outcomes related to the COVID-19 pandemic and other health outcomes within the MHS. Future analyses should evaluate the magnitude by which race/ethnicity misclassification could affect estimates of disparities in COVID-19-related outcomes within the EPICC study population.

#### MHS GENESIS

MHS GENESIS is the electronic health record that the DoD has transitioned to for all beneficiaries [89]. MHS GENESIS receives race/ethnicity data from the Service personnel offices through DEERS. As noted already in this report, DEERS does not collect race/ethnicity data for civilian dependents. Race/ethnicity for any MHS beneficiary can also be entered into a separate and distinct race/ethnicity field in MHS GENESIS at the point of care during an encounter at an MTF. However, there is currently no method to obtain this information for beneficiaries who exclusively receive care through the purchased care network. The Defense Health Board recently recommended that all beneficiaries, including civilian dependents and retirees, be able to conveniently input, view, and self-correct their race/ethnicity information both within DEERS and MHS GENESIS. They further recommended that DoD ensure that beneficiaries are provided with the relevant information to make these updates not less than annually, and that this information should be confirmed at the time of all patient check-ins.

#### Alternative Approaches

Most MDR race/ethnicity variables appear to report a single race (Supplemental Tables 1 and 2); however, the DOD_RACE_CD variable does report multiple races. Other sources of race/ethnicity data within the MDR include the Composite Health Care System, GENESIS, the Health Care Survey of DoD Beneficiaries, and TRICARE Encounter Data, among others (Supplemental Tables 1 and 2). The completeness and accuracy of this and other data sources are unknown, requiring further investigation. Given that race/ethnicity are not captured for dependent beneficiaries during DEERS enrollment, the high degree of missingness from the MDR DEERS-related table (VM6BEN) noted in these analyses is expected. Unpublished data from two large cohort studies demonstrate that alternative strategies to obtain race/ethnicity from the MDR (e.g., through use of data collected at medical encounters) may substantially reduce missingness (e.g., to ~ 45% for dependent beneficiaries; personal communication, S. Richard and B. Agan). Use of such an approach can enable further race/ethnicity-related research data among dependent beneficiaries using MDR; however, missingness may likely not be at random and further evaluation is needed to inform methodologies to address this.

### Strengths and Limitations

There are important limitations to consider within this study. First, since self-identification of race/ethnicity could vary over time, given that the EPICC race/ethnicity data was ascertained after and thus not con-current with the MDR RACE_CD and RACE_ETHNC_CD data, this may explain some MDR misclassification could. Second, we used a commonly used set of race/ethnicity variables from the MDR (i.e., “RACE_ETHNC_CD” and “RACE_CD”); however, not all MHS health disparities research is based on these same variables. Agreement and misclassification findings could differ if including other race/ethnicity variables. Further study is needed to understand concordance between other MDR race/ethnicity variables and that recorded via questionnaire. Third, there were small numbers of Asian, AI/AN, and NH/OPI participants, limiting our ability to evaluate concordance when stratified by age, sex, and branch of service in these populations so studies with oversampling of these groups need to be considered. Fourth, we did not have information regarding date of entry into the military, which hinders our ability to evaluate whether concordance varied by era. Instead, we used age as a proxy measure of era of entry into the military. Historical differences in race/ethnicity ascertainment between the branches of service could affect our findings. Additionally, there is limited written documentation on race/ethnicity ascertainment in the military historically, limiting our ability to find helpful background for this analysis and context for these findings. Finally, these findings are of limited generalizability and only applicable to the US military population and the MDR, not to other administrative databases for other healthcare or hospital systems.

There are also important strengths of this study. First, there were large counts of NH White, NH Black, and Hispanic or Latino participants. Additionally, the EPICC study questionnaire allowed participants to select as many race categories as applicable, allowing us to evaluate race/ethnicity with the most accurate information for comparison with the MDR RACE_CD and RACE_ETHNC_CD variables.

## Conclusions

There are many valuable sources of race and ethnicity data related to the MHS, including the MDR, offering the opportunity for critical health equity research within the MHS beneficiary population. Our study highlights groups within the MHS beneficiary population with a large proportion of missing race/ethnicity information as well as the potential for race/ethnicity misclassification within the MDR. With the transition to MHS GENESIS, new changes in race/ethnicity capture will be an important area of future study. To effectively identify and mitigate health disparities, including those which affect COVID-19 and future pandemic health outcomes within the MHS, further efforts to reduce misclassification and improve the completeness of race/ethnicity data is warranted.

## Supplementary Information

Below is the link to the electronic supplementary material.

Supplementary file1 (DOCX 91 KB)

## Data Availability

The data that support the findings of this study are available from the corresponding author upon reasonable request.

## Abbreviations

AI/AN: American Indian and Alaskan Native
A/PI: Asian/Pacific Islander
DEERS: Defense Enrollment Eligibility Reporting System
DMDC: Defense Manpower Data Center
DMSS: Defense Medical Surveillance System
DoDD: Department of Defense Directive
DoDI: Department of Defense Instruction
DoDSR: Department of Defense Serum Repository
EPICC: Epidemiology, Immunology, and Clinical Characteristics of Emerging Infectious Diseases with Pandemic Potential
MDR: Military Health System Data Repository
ME/NA: Middle Eastern or North African
MEPS: Military Entrance Processing Stations
MHS: Military Health System
MTF: Military treatment facility
NH: Not Hispanic or Latino
NH/OPI: Native Hawaiian or Other Pacific Islander
OMB: Office of Management and Budget
PPV: Positive predictive value
VA: Department of Veterans Affairs

## Declarations

### Disclaimer

All opinions expressed here are the authors’ and do not necessarily reflect the policies and official views of the Uniformed Services University of the Health Sciences or the Department of Defense. The contents of this publication are the sole responsibility of the author(s) and do not necessarily reflect the views, opinions, or policies of Henry M. Jackson Foundation for the Advancement of Military Medicine, Inc. (HJF), Uniformed Services University of the Health Sciences (USUHS), the Department of Defense (DoD), the Departments of the Army, Navy, or Air Force, or the Defense Health Agency. Mention of trade names, commercial products, or organizations does not imply endorsement by the US Government.

CHO, SC, CB, RO, THB, DRT, JDM and JAR are service members or employees of the US Government. This work was prepared as part of their official duties. Title 17 U.S.C. §105 provides that “Copyright protection under this title is not available for any work of the United States Government.” Title 17 U.S.C. §101 defines a U.S. Government work as a work prepared by a military service member or employee of the US Government as part of that person’s official duties.

### The following EPICC Study Team Members are Listed as Group Authors for this Paper

Brooke Army Medical Center, Fort Sam Houston, TX: T. Merritt

Naval Medical Center San Diego, San Diego, CA: C. Berjohn, Ryan C. Maves.

Uniformed Services University of the Health Sciences, Bethesda, MD: M. Fritschlanski

United States Air Force School of Aerospace Medicine, Dayton, OH: R. Chapleau
