# Supplementary material for: Statistical Accuracy of Administratively Recorded Race/Ethnicity in the Military Health System and Race/Ethnicity Ascertained via Questionnaire
Source: J Racial Ethn Health Disparities. 2025 Mar 21;13(2):1513–26. doi: 10.1007/s40615-025-02351-7 (PMC12966218; doi:10.1007/s40615-025-02351-7)
Supplement: Supplementary file 1 — Supplementary file1 (DOCX 91 KB) [file 40615_2025_2351_MOESM1_ESM.docx]

**SUPPLEMENTAL Material**

**Supplemental Figure 1.**


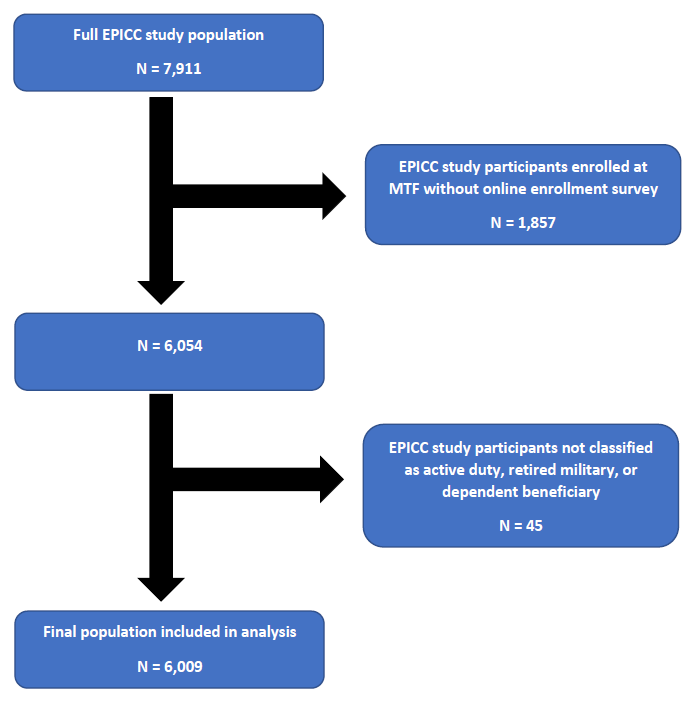


**Flow chart of the inclusion–exclusion process.**

**Supplemental Table 1**. Summary of race information within the MDR.

| **Source** | **MDR File Name(s)** | **Data Element Name** | **SAS Variable Name or Position** | **Values or categories** |
| --- | --- | --- | --- | --- |
| MDR VM6 | - VM6BEN | DoD Race Code | DOD_RACE_CD | 001=American Indian/Alaska Native  002=Asian (All but USAR include Pacific Islander in this Code)  003=Black or African American  004=Native Hawaiian or other Pacific Islander (USAR submits F for Pacific Islander)  005=White  100=American Indian/Alaska Native, Asian  101=American Indian/Alaska Native, Asian, Black or African American  102=American Indian/Alaska Native, Asian, Black or African American, Native Hawaiian or other Pacific Islander  103=American Indian/Alaska Native, Asian, Black or African American, Native Hawaiian or other Pacific Islander, White  104=American Indian/Alaska Native, Asian, Black or African American, White  105=American Indian/Alaska Native, Asian, Native Hawaiian or other Pacific Islander  106=American Indian/Alaska Native, Asian, Native Hawaiian or other Pacific Islander, White  107=American Indian/Alaska Native, Asian, White  108=American Indian/Alaska Native, Black or African American  109=American Indian/Alaska Native, Black or African American, Native Hawaiian or other Pacific Islander  110=American Indian/Alaska Native, Black or African American, Native Hawaiian or other Pacific Islander, White  111=American Indian/Alaska Native, Black or African American, White  112=American Indian/Alaska Native, Native Hawaiian or other Pacific Islander  113=American Indian/Alaska Native, Native Hawaiian or other Pacific Islander, White  114=American Indian/Alaska Native, White  115=Asian, Black or African American  116=Asian, Black or African American, Native Hawaiian or other Pacific Islander  117=Asian, Black or African American, Native Hawaiian or other Pacific Islander, White  118=Asian, Black or African American, White  119=Asian, Native Hawaiian or other Pacific Islander  120=Asian, Native Hawaiian or other Pacific Islander, White  121=Asian, White  122=Black or African American, Native Hawaiian or other Pacific Islander  123=Black or African American, Native Hawaiian or other Pacific Islander, White  124=Black or African American, White  125=Native Hawaiian or other Pacific Islander, White  999=Other  ZZZ=Unknown |
| CHCS/ADS (*Composite Health Care System and Ambulatory Data System*) | - CAPER (Basic) - CAPER (Enhanced) | Patient Race Code | PATRACE | C = White  M = Asian or Pacific Islander  N = Black  R = Western Hemisphere Indians  X = Other  Z = Unknown |
|  | - SADR - SIDR | Race |  |  |
| Military Medical Support Office | - JOES C | What is your race? White | QRACEv1_1 | 0=Not White  1=White |
|  |  | What is your race? Black or African American | QRACEv1_2 | 0=Not Black or African American  1=Black or African American |
|  |  | What is your race? Asian | QRACEv1_3 | 0=Not Asian  1=Asian |
|  |  | What is your race? Native Hawaiian or other Pacific Islander | QRACEv1_4 | 0=Not Native Hawaiian or other Pacific Islander  1=Native Hawaiian or other Pacific Islander |
|  |  | What is your race? American Indian or Alaskan Native | QRACEv1_5 | 0=Not American Indian or Alaskan Native  1=American Indian or Alaskan Native |
| MHS GENESIS BDE 2.4 and 3.0 | - GENESIS Admission (BDE 3.0) - GENESIS Episodic Enc (BDE 2.4 and 3.0) | DEERS Patient Race Code | RACE | C = White  M = Asian or Pacific Islander  N = Black  R = American Indian or Alaskan Native  X = Other  Z = Unknown |
| DEERS (*Defense Enrollment Eligibility Reporting System*) | - DESPROV (Clinical) - Direct Care Dental Encounter Data | DEERS Race Code |  |  |
| MHS GENESIS BDE 2.4 and 3.0 | - GENESIS Radiology (BDE 2.4 and 3.0) - GENESIS Surgery |  |  |  |
| TED Database | - TED-I Header - TED-NI |  |  |  |
| TDP2 contractor (MetLife), TDP3 contractor (UCCI) | - TDP Claims |  |  |  |
| TRDP Contractor (*TRICARE Retiree Dental Program*) -- Delta Dental | - TRDP |  |  |  |
| MHS GENESIS BDE 2.4 and 3.0 | - GENESIS Lab Results (BDE 2.4 and 3.0) - GENESIS Laboratory (BDE 2.4 and 3.0) - GENESIS Microbio Res (BDE 2.4 and 3.0) | Patient Race |  |  |
| MHS GENESIS BDE 2.4 and 3.0 | - GENESIS Person (BDE 2.4 and 3.0) | Person Race |  |  |
| GENESIS (Cerner Millennium) | - GENESIS Immunization table | Race |  |  |
| MHS GENESIS BDE 2.4 and 3.0 | - GENESIS Appointment (BDE 2.4 and 3.0) - GENESIS Referral (BDE 2.4 and 3.0) |  |  |  |
|  | - GENESIS Pharmacy (BDE 2.4 and 3.0) | Race Code |  |  |
|  | - GENESIS Vitals (BDE 2.4 and 3.0) | Race of Record |  |  |
| MHS GENESIS BDE 2.4 | - TRDP - GENESIS Admission (BDE 2.4) - GENESIS Basic Enc (BDE 2.4) | DEERS Patient Race Code | RACE | A = American Indian/Alaskan Native  B = Asian  D = Native Hawaiian or Pacific Islander  E = White  G = Black or African American  X = Other  Z = Unknown |
| MDR PITE (*MHS Data Repository Point in Time Extract*) | - LVM4 - LVM6 | Race | RACE_CD | C = White  M = Asian or Pacific Islander  N = Black  R = American Indian or Alaskan native  X = Other  Z = Unknown |
| MDR PITE (*MHS Data Repository Point in Time Extract*) | - PBEN - VM4BEN | Race Code |  |  |
| MDR VM6 | - VM6BEN | Race Code* |  |  |
| MHS GENESIS BDE 3.0 | - Race Code DEERS | Race Code DEERS | RACE_D | C = White  M = Asian or Pacific Islander  N = Black  R = American Indian or Alaskan native  X = Other  Z = Unknown |
|  | - GENESIS Episodic Enc (BDE 3.0) | DEERS Patient Race Code | RACE_DEERS | C = White  M = Asian or Pacific Islander  N = Black  R = American Indian or Alaskan native  X = Other  Z = Unknown |
| HCSDB (*Health Care Survey of DoD Beneficiaries*) | - HCSDB | Race/Ethnic Code | RACEETHN | A = American Indian or Alaskan Native  B = Asian or Pacific islander  C = Black (not Hispanic)  D = White (not Hispanic)  E = Hispanic  X = Other  Z = Unknown |
| DEERS (*Defense Enrollment Eligibility Reporting System*) | - VM4AGG - VM6PAGG | Race/Ethnicity |  |  |
| MDR PITE (*MHS Data Repository Point in Time Extract*) | - PAGG |  |  |  |
| MDR PITE (*MHS Data Repository Point in Time Extract*) | - PBEN - VM4BEN | Race Ethnic Code | RACE_ETHNC_CD | A = American Indian/Alaskan Native  B = Asian or Pacific Islander  C = Black, not Hispanic  D = white, not Hispanic  E = Hispanic  X = Other  Z = Unknown |
| MDR VM6 | - VM6BEN | Race Ethnic Code* |  |  |
| MHS GENESIS BDE 3.0 | - GENESIS Pharmacy (BDE 3.0) | Race (derived) | RACE_G | American Indian or Alaska Native  Asian  Black or African American  Native Hawaiian or Pacific Islander  Other Race  White  blank |
| MHS GENESIS BDE 3.0 | - GENESIS Episodic Enc (BDE 3.0) | Patient Race Code GENESIS | RACE_GENESIS | American Indian or Alaska Native  Asian  Black or African American  Native Hawaiian or Pacific Islander  Other Race  White |
| PEO DHCS (*Program Executive Office Defense Health Clinical Systems*) | - TMDS People (Basic) | Race Id | RACE_ID | - |
| MHS GENESIS BDE 3.0 | - GENESIS Orders (BDE 2.4 and 3.0) | Race | RACE_R | American Indian or Alaska Native  Asian  Black or African American  Native Hawaiian or Other Pacific Islander  Other Race  White |
| MHS GENESIS BDE 2.4 | - GENESIS Referral (BDE 2.4) |  |  |  |
| MHS GENESIS BDE 2.4 and BDE 3.0 | - GENESIS Surgery | Race of Record |  |  |
| HCSDB (*Health Care Survey of DoD Beneficiaries*) | - HCSDB | Race: White | SRRACEA | 1=marked  2=not marked |
| HCSDB (*Health Care Survey of DoD Beneficiaries*) | - HCSDB | Race: Black or African American | SRRACEB | 1=marked  2=not marked |
| HCSDB (*Health Care Survey of DoD Beneficiaries*) | - HCSDB | Race: American Indian or Alaska Native | SRRACEC | 1=marked  2=not marked |
| HCSDB (*Health Care Survey of DoD Beneficiaries*) | - HCSDB | Race: Asian | SRRACED | 1=marked  2=not marked |
| HCSDB (*Health Care Survey of DoD Beneficiaries*) | - HCSDB | Race: Native Hawaiian/other Pacific Isl. | SRRACEE | 1=marked  2=not marked |
| HCSDB (*Health Care Survey of DoD Beneficiaries*) | - HCSDB | Race: Other | SRRACEF | 1=marked  2=not marked |

* Data dictionary notes “Race and ethnicity of beneficiary. This variable is unreliable for non-sponsors. Users should not assume that the race/ethnicity of a sponsor is the same as the race of a family member.”

Abbreviations: ADS = Ambulatory Data System; CAPER = Comprehensive Ambulatory/Professional Encounter Record; CHS = Composite Health Care System; DEERS = Defense Enrollment Eligibility Reporting System; DESPROV = Designated Provider File; DoD = Department of Defense; HCSDB = Health Care Survey of DoD Beneficiaries; JOES-C = Joint Outpatient Experience Survey – Consumer Assessment of Health Providers and Systems; LVM4 = Longitudinal VM4; LVM6 = Longitudinal VM6; MDR = MHS Data Repository; MHS = Military Health System; PAGG = PITE Aggregate file; PBEN = PITE Beneficiary level; PEO DHCS = Program Executive Office Defense Health Clinical Systems; PITE = Point in Time Extract; SADR = Standard Ambulatory Data Record; SIDR = Standard Inpatient Data Record; TDP = Tricare Dental Program; TED-I = TRICARE Encounter Data – Institutional; TED-NI = TRICARE Encounter Data—Non-institutional; TMDS = Theater Medical Data Store; TRDP = TRICARE Retiree Dental Program; VM4AGG = VM4 Aggregate File; VM4BEN = VM4 Beneficiary Level; VM6BEN = VM6 Beneficiary Level; VM6PAGG = PITE Aggregate File

**Supplemental Table 2**. Descriptions of ethnicity information within the MDR.

| **Source** | **MDR File Name(s)** | **Data Element Name** | **SAS Variable Name** | **Ethnicity categories** |
| --- | --- | --- | --- | --- |
| MDR PITE (*Point in Time Extract*) | - LVM4 - LVM6 | Ethnicity | ETHNC_NAT_ORIG_CD | 1 = Other Hispanic  2 = US or Canadian Indian Tribe  3 = Other Asian  4 = Puerto Rican  5 = Filipino  6 = Mexican  7 = Eskimo  8 = Aleut  9 = Cuban  D = Indian  E = Melanesian  G = Chinese  H = Guamanian  J = Japanese  K = Korean  L = Polynesian  Q = Pacific Islander NEC  S = Latin America w/ Hispanic descent  V = Vietnamese  W = Micronesian  X = Other  Y = None  Z = Unknown |
|  | - PBEN - VM4BEN | Ethnicity National Origin Code |  |  |
| MDR VM6 | - VM6BEN |  |  |  |
| CHCS/ADS (*Composite Health Care System and Ambulatory Data System*) | - SIDR | Ethnic Classification | ETHNIC | 1 = Hispanic  2 = SE Asian  3 = Filipino  4 = Other Asian Pacific Islander  9 = Other  Z = Unknown |
| DEERS (*Defense Enrollment Eligibility Reporting System*) | - DESPROV (Clinical) - Direct Care Dental Encounter Data - GENESIS Admission (BDE 2.4) | DEERS Ethnicity Code | ETHNIC | 1 = Other Hispanic  2 = US or Canadian Indian Tribe  3 = Other Asian  4 = Puerto Rican  5 = Filipino  6 = Mexican  7 = Eskimo  8 = Aleut  9 = Cuban  D = Indian  E = Melanesian  G = Chinese  H = Guamanian  J = Japanese  K = Korean  L = Polynesian  Q = Pacific Islander NEC  S = Latin America w/ Hispanic descent  V = Vietnamese  W = Micronesian  X = Other  Y = None  Z = Unknown |
| MHS GENESIS BDE 2.4 | - GENESIS Radiology (BDE 2.4) |  |  |  |
| TDP2 contractor (MetLife), TDP3 contractor (UCCI) | - TDP Claims |  |  |  |
| TED (*TRICARE Encounter Data*) Database | - TED-I Header - TED-NI |  |  |  |
| TRDP (*TRICARE Retiree Dental Program*) Contractor (Delta Dental) | - TRDP |  |  |  |
| MHS GENESIS BDE 2.4 | - GENESIS Admission (BDE 2.4) - GENESIS Basic Enc (BDE 2.4) | Ethnic Background Code |  |  |
| MHS GENESIS BDE 3.0 | - GENESIS Admission (BDE 3.0) | Ethnic Group |  |  |
| GENESIS (Cerner Millennium) | - GENESIS Immunization table |  |  |  |
| MHS GENESIS BDE 2.4 and BDE 3.0 | - GENESIS Surgery |  |  |  |
| MHS GENESIS BDE 2.4 and 3.0 | - MHS Genesis Appointment (BDE 2.4) - GENESIS Referral (BDE 2.4 and 3.0) |  |  |  |
| MHS GENESIS BDE 2.4 | - GENESIS Vitals (BDE 2.4) | Ethnic Group of Record |  |  |
| MHS GENESIS BDE 2.4 | - GENESIS Pharmacy (BDE 2.4) | Ethnicity Code |  |  |
| MHS GENESIS BDE 3.0 | - GENESIS Pharmacy (BDE 3.0) |  |  |  |
| MHS GENESIS BDE 2.4 | - GENESIS Laboratory (BDE 2.4) - GENESIS Lab Results (BDE 2.4) - GENESIS Microbio Res (BDE 2.4) | Patient Ethnicity |  |  |
| MHS GENESIS BDE 3.0 | - GENESIS Radiology (BDE 3.0) | DEERS Ethnicity Code | ETHNIC | H = Hispanic or Latino  N = Not Hispanic or Latino |
|  | - GENESIS Admission (BDE 3.0) - GENESIS Episodic Enc (BDE 3.0) | Ethnic Background Code |  |  |
| MHS GENESIS BDE 3.0 | - GENESIS Laboratory (BDE 3.0) - GENESIS Lab Results (BDE 3.0) | Patient Ethnicity |  |  |
| MHS GENESIS BDE 2.4 and 3.0 | - GENESIS Person (BDE 2.4 and 3.0) |  |  |  |
| CHCS/ADS (*Composite Health Care System and Ambulatory Data System*) | - CAPER (Basic) - CAPER (Enhanced) | Ethnic Background | ETHNICGR | 1 = Hispanic  2 = SE Asian  3 = Filipino  4 = Other Asian Pacific Islander  9 = Other  Z = Unknown |
|  | - SADR | Ethnic Group |  |  |
| MHS GENESIS BDE 3.0 | - GENESIS Pharmacy (BDE 3.0) | Ethnicity Code DEERS | ETHNIC_D | 1 = Other Hispanic  2 = US or Canadian Indian Tribe  3 = Other Asian  4 = Puerto Rican  5 = Filipino  6 = Mexican  7 = Eskimo  8 = Aleut  9 = Cuban  D = Indian  E = Melanesian  G = Chinese  H = Guamanian  J = Japanese  K = Korean  L = Polynesian  Q = Pacific Islander NEC  S = Latin America w/ Hispanic descent  V = Vietnamese  W = Micronesian  X = Other  Y = None  Z = Unknown |
| MHS GENESIS BDE 3.0 | - GENESIS Episodic Enc (BDE 3.0) | Ethnic Background Code—DEERS | ETHNIC_DEERS | 1 = Other Hispanic  2 = US or Canadian Indian Tribe  3 = Other Asian  4 = Puerto Rican  5 = Filipino  6 = Mexican  7 = Eskimo  8 = Aleut  9 = Cuban  D = Indian  E = Melanesian  G = Chinese  H = Guamanian  J = Japanese  K = Korean  L = Polynesian  Q = Pacific Islander NEC  S = Latin America w/ Hispanic descent  V = Vietnamese  W = Micronesian  X = Other  Y = None  Z = Unknown |
| MHS GENESIS BDE 2.4 | - GENESIS Orders (BDE 2.4) | Ethnicity | ETHNIC_R | H = Hispanic  N = Non-Hispanic  blank = Unknown |
| MHS GENESIS BDE 2.4 and BDE 3.0 | - GENESIS Surgery | Ethnic Group of Record |  |  |
| MHS GENESIS BDE 2.4 and 3.0 | - MHS Genesis Appointment (BDE 2.4) - GENESIS Admission (BDE 3.0) - GENESIS Referral (BDE 2.4 and 3.0) - GENESIS Vitals (BDE 3.0) |  |  |  |
| HCSDB (*Health Care Survey of DoD Beneficiaries*) | - HCSDB | Race/Ethnic Code | RACEETHN | A = American Indian or Alaskan Native  B = Asian or Pacific islander  C = Black (not Hispanic)  D = White (not Hispanic)  E = Hispanic  X = Other  Z = Unknown |
| MDR PITE (*Point in Time Extract*) | - PAGG | Race/Ethnicity |  |  |
| DEERS (*Defense Enrollment Eligibility Reporting System*) | - VM4AGG |  |  |  |
| MDR PITE (*Point in Time Extract*) | - PBEN - VM4BEN | Race Ethnic Code | RACE_ETHNC_CD | A = American Indian or Alaskan Native  B = Asian or Pacific islander  C = Black (not Hispanic)  D = White (not Hispanic)  E = Hispanic  X = Other  Z = Unknown |
| MDR VM6 | - VM6BEN | Race Ethnic Code* |  |  |

* Data dictionary notes “Race and ethnicity of beneficiary. This variable is unreliable for non-sponsors. Users should not assume that the race/ethnicity of a sponsor is the same as the race of a family member.”

Abbreviations: ADS = Ambulatory Data System; CAPER = Comprehensive Ambulatory/Professional Encounter Record; CHS = Composite Health Care System; DEERS = Defense Enrollment Eligibility Reporting System; DESPROV = Designated Provider File; DoD = Department of Defense; HCSDB = Health Care Survey of DoD Beneficiaries; JOES-C = Joint Outpatient Experience Survey – Consumer Assessment of Health Providers and Systems; LVM4 = Longitudinal VM4; LVM6 = Longitudinal VM6; MDR = MHS Data Repository; MHS = Military Health System; PAGG = PITE Aggregate file; PBEN = PITE Beneficiary level; PEO DHCS = Program Executive Office Defense Health Clinical Systems; PITE = Point in Time Extract; SADR = Standard Ambulatory Data Record; SIDR = Standard Inpatient Data Record; TDP = Tricare Dental Program; TED-I = TRICARE Encounter Data – Institutional; TED-NI = TRICARE Encounter Data—Non-institutional; TMDS = Theater Medical Data Store; TRDP = TRICARE Retiree Dental Program; VM4AGG = VM4 Aggregate File; VM4BEN = VM4 Beneficiary Level; VM6BEN = VM6 Beneficiary Level; VM6PAGG = PITE Aggregate File

**Supplemental Table 3.** Descriptions of the statistical measures employed.

| **Measure** | **Description** | **Calculation in a 2x2 table** |
| --- | --- | --- |
| Sensitivity | The proportion of participants identified as a particular race based on EPICC questionnaire responses who are correctly recorded as that race in the MDR. | sensitivity = [(true positives)/(true positives + false negatives)] x 100 |
| Positive Predictive Value (PPV) | The proportion of patients recorded as a particular race/ethnicity in the MDR who identified as that race via the EPICC questionnaire. | PPV = [(true positives)/(true positives + false positives)] x 100 |
| Cohen’s Kappa | Used for interrater or intra-rater reliability testing in 2x2 tables, it can range from -1.0 to +1.0 An estimate of 0 represents the amount of agreement expected from random chance, and +1.0 represents perfect agreement between the MDR and EPICC [1]. | -- |
| Simple Kappa | A special case of Cohen’s kappa in which all observations are classified by the same two raters (in this case MDR and EPICC) and no weighting is used. | -- |

Note: EPICC questionnaire responses were considered the gold standard.

**Supplemental Table 4.** Counts, sensitivity, PPV, and Cohen’s kappa coefficients by race/ethnicity category comparing MDR and EPICC questionnaire data, excluding missing/unknown data.

| **Active Duty + Retired Military (n=5609)** | | | | | | | |
| --- | --- | --- | --- | --- | --- | --- | --- |
|  |  | **EPICC Questionnaire** | | | | | |
|  |  | **NH White (n=3491)** | **NH Black (n=608)** | **Hispanic or Latino (any race) (n=1097)** | **NH A/PI (n=332)** | **NH AI/AN (n=23)** | **NH Other (n=58)** |
| **MDR** | **NH White (n=3487)** | N=3308  C (sen): 94.76%  R (PPV): 94.87%  CK: 0.86 | N=4  C: 0.66%  R: 0.11% | N=143  C: 13.04%  R: 4.10% | N=7  C: 2.11%  R: 0.20% | N=6  C: 26.09%  R: 0.17% | N=19  C: 32.76%  R: 0.54% |
|  | **NH Black (n=603)** | N=3  C: 0.09%  R: 0.50% | N=560  C (sen): 92.11%  R (PPV): 92.87%  CK: 0.92 | N=18  C: 1.64%  R: 2.99% | N=0 | N=1  C: 4.35%  R: 0.17% | N=21  C: 36.21%  R: 3.48% |
|  | **Hispanic or Latino (any race) (n=954)** | N=36  C: 1.03%  R: 3.77% | N=6  C: 0.99%  R: 0.63% | N=892  C (sen): 81.31%  R (PPV): 93.50%  CK: 0.84 | N=10  C: 3.01%  R: 1.05% | N=1  C: 4.35%  R: 0.10% | N=9  C: 15.52%  R: 0.94% |
|  | **NH A/PI (n=299)** | N=9  C: 0.26%  R: 3.01% | N=0 | N=7  C: 0.64%  R: 2.34% | N=279  C (sen): 84.04%  R (PPV): 93.31%  CK: 0.88 | N=0 | N=4  C: 6.90%  R: 1.34% |
|  | **NH AI/AN (n=30)** | N=9  C: 0.26%  R: 30.00% | N=0 | N=7  C: 0.64%  R: 23.33% | N=0 | N=14  C (sen): 60.87%  R (PPV): 46.67%  CK: 0.53 | N=0 |
|  | **NH Other (n=236)** | N=126  C: 3.61%  R: 53.39% | N=38  C: 6.25%  R: 16.10% | N=30  C: 2.73%  R: 12.71% | N=36  C: 10.84%  R: 15.25% | N=1  C: 4.35%  R: 0.42% | N=5  C (sen): 8.62%  R (PPV): 2.12%  CK: 0.02 |
| **Dependent Beneficiaries (n=329)** | | | | | | | |
|  |  | **EPICC Questionnaire** | | | | | |
|  |  | **NH White (n=186)** | **NH Black (n=46)** | **Hispanic or Latino (any race) (n=72)** | **NH A/PI (n=25)** | **NH AI/AN (n=0)** | **NH Other (n=0)** |
| **MDR** | **NH White (n=217)** | N=182  C (sen): 97.85%  R (PPV): 83.87%  CK: 0.75 | N=4  C: 8.70%  R: 1.84% | N=27  C: 37.50%  R: 12.44% | N=4  C: 16.00%  R: 1.84% | N=0 | N=0 |
|  | **NH Black (n=47)** | N=2  C: 1.08%  R: 4.26% | N=42  C (sen): 91.30%  R (PPV): 89.36%  CK: 0.89 | N=3  C: 4.17%  R: 6.38% | N=0 | N=0 | N=0 |
|  | **Hispanic or Latino (any race) (n=26)** | N=0 | N=0 | N=26  C (sen): 36.11%  R (PPV): 100.00%  CK: 0.47 | N=0 | N=0 | N=0 |
|  | **NH A/PI (n=21)** | N=0 | N=0 | N=4  C: 5.56%  R: 19.05% | N=17  C (sen): 68.00%  R (PPV): 80.95%  CK: 0.72 | N=0 | N=0 |
|  | **NH AI/AN (n=2)** | N=0 | N=0 | N=2  C: 2.78%  R: 100.00% | -- | N=0 | N=0 |
|  | **NH Other (n=16)** | N=2  C: 1.08%  R: 12.50% | N=0 | N=10  C: 13.89%  R: 62.50% | N=4  C: 16.00%  R: 25.00% | N=0 | N=0 |

Note: Shading indicates matched race/ethnicity categories. Row percent (R) is equivalent to positive predictive value and column percent (C) is equivalent to sensitivity. Participants indicating ‘Prefer not to answer’ for race in EPICC are excluded from these analyses. ‘MDR’ refers to the MDR’s RACE_ETHNC_CD variable.

Abbreviations: AI/AN = American Indian/Alaskan Native; A/PI = Asian/Pacific Islander; C = column percent; CK = Cohen’s kappa coefficient; MDR EPICC = The Epidemiology, Immunology, and Clinical Characteristics of Emerging Infectious Diseases with Pandemic Potential (EPICC) study; MDR = Military Health System Data Repository; NH = non-Hispanic; PPV = positive predictive value; R = row percent; sen = sensitivity

**Supplemental Table 5.** Counts, sensitivity, PPV, and Cohen’s kappa coefficients by race category comparing MDR and EPICC questionnaire data, including missing/unknown data.

| **Active Duty + Retired Military (n=6048)** | | | | | | | |
| --- | --- | --- | --- | --- | --- | --- | --- |
|  |  | **EPICC Questionnaire** | | | | | |
|  |  | **White (n=4303)** | **Black (n=689)** | **A/PI (n=354)** | **AI/AN (n=51)** | **Other (n=247)** | **Missing (n=404)** |
| **MDR** | **White (n=4379)** | N=3942  C (sen): 91.6%  R (PPV): 90.0%  CK: 0.67 | N=12  C: 1.7%  R: 0.3% | N=13  C: 3.7%  R: 0.3% | N=19  C: 37.3%  R: 0.4% | N=150  C: 60.7%  R: 3.4% | N=243  C: 60.2%  R: 5.6% |
|  | **Black (n=722)** | N=5  C: 0.1%  R: 0.7% | N=602  C (sen): 87.4%  R (PPV): 83.4%  CK: 0.83 | N=0 | N=2  C: 3.9%  R: 0.3% | N=29  C: 11.7%  R: 4.0% | N=84  C: 20.8%  R: 11.6% |
|  | **A/PI (n=372)** | N=22  C: 0.5%  R: 5.9% | N=2  C: 0.3%  R: 0.5% | N=294  C (sen): 83.1%  R (PPV): 79.0%  CK: 0.80 | N=2  C: 3.9%  R: 0.5% | N=14  C: 5.7%  R: 3.8% | N=38  C: 9.4%  R: 10.2% |
|  | **AI/AN (n=50)** | N=16  C: 0.4%  R: 32.0% | N=2  C: 0.3%  R: 4.0% | N=0 | N=24  C (sen): 47.1%  R (PPV): 48.0%  CK: 0.47 | N=4  C: 1.6%  R: 8.0% | N=4  C: 1.0%  R: 8.0% |
|  | **Other (n=361)** | N=197  C: 4.6%  R: 54.6% | N=44  C: 6.4%  R: 12.2% | N=40  C: 11.3%  R: 11.1% | N=3  C: 5.9%  R: 0.8% | N=45  C (sen): 18.2%  R (PPV): 12.5%  CK: 0.10 | N=32  C: 7.9%  R: 8.9% |
|  | **Missing/**  **Unknown (n=164)** | N=121  C: 2.8%  R: 73.8% | N=27  C: 3.9%  R: 16.5% | N=7  C: 2.0%  R: 4.3% | N=1  C: 2.0%  R: 0.6% | N=5  C: 2.0%  R: 3.1% | N=3  C (sen): 0.7%  R (PPV): 1.8%  CK: -0.03 |
| **Dependent Beneficiaries (n=1196)** | | | | | | | |
|  |  | **EPICC Questionnaire** | | | | | |
|  |  | **White (n=901)** | **Black (n=123)** | **A/PI (n=97)** | **AI/AN (n=10)** | **Other (n=47)** | **Missing (n=18)** |
| **MDR** | **White (n=235)** | N=213  C (sen): 23.6%  R (PPV): 90.6%  CK: 0.09 | N=5  C: 4.1%  R: 2.1% | N=4  C: 4.1%  R: 1.7% | N=1  C: 10.0%  R: 0.4% | N=7  C: 14.9%  R: 3.0% | N=5  C: 27.8%  R: 2.1% |
|  | **Black (n=49)** | N=2  C: 0.2%  R: 4.1% | N=44  C (sen): 35.8%  R (PPV): 89.8%  CK: 0.48 | N=0 | N=0 | N=1  C: 2.1%  R: 2.0% | N=2  C: 11.1%  R: 4.1% |
|  | **A/PI (n=23)** | N=5  C: 0.6%  R: 21.7% | N=0 | N=17  C (sen): 17.5%  R (PPV): 73.9%  CK: 0.26 | N=0 | N=0 | N=1  C: 5.6%  R: 4.4% |
|  | **AI/AN (n=3)** | N=2  C: 0.2%  R: 66.7% | N=0 | N=0 | N=0 | N=1  C: 2.1%  R: 33.3% | N=0 |
|  | **Other (n=22)** | N=15  C: 1.7%  R: 68.2% | N=0 | N=4  C: 4.1%  R: 18.2% | N=0 | N=3  C (sen): 6.4%  R (PPV): 13.6%  CK: 0.06 | N=0 |
|  | **Missing/**  **Unknown (n=864)** | N=664  C: 73.7%  R: 76.9% | N=74  C: 60.2%  R: 8.6% | N=72  C: 74.2%  R: 8.3% | N=9  C: 90.0%  R: 1.0% | N=35  C: 74.5%  R: 4.1% | N=10  C (sen): 55.6%  R (PPV): 1.2%  CK: -0.01 |

Notes: Shading indicates matched race categories. Row percent (R) is equivalent to positive predictive value and column percent (C) is equivalent to sensitivity. Participants indicating ‘Prefer not to answer’ for race in EPICC are excluded from these analyses. ‘MDR’ refers to the MDR’s RACE_CD variable.

Abbreviations: AI/AN = American Indian/Alaskan Native; A/PI = Asian/Pacific Islander; C = column percent; CK = Cohen’s Kappa coefficient; EPICC = The Epidemiology, Immunology, and Clinical Characteristics of Emerging Infectious Diseases with Pandemic Potential (EPICC) study; MDR = Military Health System Data Repository; PPV = positive predictive value; R = row percent; sen = sensitivity

**Supplemental Table 6.** Counts, sensitivity, PPV, and Cohen’s kappa coefficients by race category comparing MDR and EPICC questionnaire data, excluding missing/unknown data.

| **Active Duty + Retired Military (n=5483)** | | | | | | | | | | |
| --- | --- | --- | --- | --- | --- | --- | --- | --- | --- | --- |
|  |  | **EPICC Questionnaire** | | | | | | | | |
|  |  | **White (n=4182)** | | **Black (n=662)** | | **A/PI (n=347)** | | **AI/AN (n=50)** | | **Other (n=242)** |
| **MDR** | **White (n=4136)** | N=3942  C (sen): 94.26%  R (PPV): 95.31%  CK: 0.78 | | N=12  C: 1.81%  R: 0.29% | | N=13  C: 3.75%  R: 0.31% | | N=19  C: 38.00%  R: 0.46% | | N=150  C: 61.98%  R: 3.63% |
|  | **Black (n=638)** | N=5  C: 0.12%  R: 0.78% | | N=602  C (sen): 90.94%  R (PPV): 94.36%  CK: 0.92 | | N=0 | | N=2  C: 4.00%  R: 0.31% | | N=29  C: 11.98%  R: 4.55% |
|  | **A/PI (n=334)** | N=22  C: 0.53%  R: 6.59% | | N=2  C: 0.30%  R: 0.60% | | N=294  C (sen): 84.73%  R (PPV): 88.02%  CK: 0.85 | | N=2  C: 4.00%  R: 0.60% | | N=14  C: 5.79%  R: 4.19% |
|  | **AI/AN (n=46)** | N=16  C: 0.38%  R: 34.78% | | N=2  C: 0.30%  R: 4.35% | | N=0 | | N=24  C (sen): 48.00%  R (PPV): 52.17%  CK: 0.50 | | N=4  C: 1.65%  R: 8.70% |
|  | **Other (n=329)** | N=197  C: 4.71%  R: 59.88% | | N=44  C: 6.65%  R: 13.37% | | N=40  C: 11.53%  R: 12.16% | | N=3  C: 6.00%  R: 0.91% | | N=45  C (sen): 18.60%  R (PPV): 13.68%  CK: 0.11 |
| **Dependent Beneficiaries (n=324)** | | | | | | | | | | |
|  |  | **EPICC Questionnaire** | | | | | | | | |
|  |  | **White (n=237)** | **Black (n=49)** | | **A/PI (n=25)** | | **AI/AN (n=1)** | | **Other (n=12)** | |
| **MDR** | **White (n=230)** | N=213  C (sen): 89.87%  R (PPV): 92.61%  CK: 0.69 | N=5  C: 10.20%  R: 2.17% | | N=4  C: 16.00%  R: 1.74% | | N=1  C: 100.0%  R: 0.43% | | N=7  C: 58.33%  R: 3.04% | |
|  | **Black (n=47)** | N=2  C: 0.84%  R: 4.26% | N=44  C (sen): 89.80%  R (PPV): 93.62%  CK: 0.90 | | N=0 | | N=0 | | N=1  C: 8.33%  R: 2.13% | |
|  | **A/PI (n=22)** | N=5  C: 2.11%  R: 22.73% | N=0 | | N=17  C (sen): 68.00%  R (PPV): 77.27%  CK: 0.70 | | N=0 | | N=0 | |
|  | **AI/AN (n=3)** | N=2  C: 0.22%  R: 66.67% | N=0 | | N=0 | | N=0 | | N=1  C: 8.33%  R: 33.33% | |
|  | **Other (n=22)** | N=15  C: 6.33%  R: 68.18% | N=0 | | N=4  C: 16.00%  R: 18.18% | | N=0 | | N=3  C (sen): 25.00%  R (PPV): 13.64%  CK: 0.14 | |

Note: Shading indicates matched race/ethnicity categories. Row percent (R) is equivalent to positive predictive value and column percent (C) is equivalent to sensitivity. Participants indicating ‘Prefer not to answer’ for race in EPICC are excluded from these analyses. ‘MDR’ refers to the MDR’s RACE_CD variable.

Abbreviations: AI/AN = American Indian/Alaskan Native; A/PI = Asian/Pacific Islander; C = column percent; CK = Cohen’s kappa coefficient; EPICC = The Epidemiology, Immunology, and Clinical Characteristics of Emerging Infectious Diseases with Pandemic Potential (EPICC) study; MDR = Military Health System Data Repository; PPV = positive predictive value; R = row percent; sen = sensitivity

**Supplemental Table 7**. Counts of write-in responses for participants indicating ‘Other’ race^1^ in the EPICC questionnaire (n=294).

| **Description** | **N (%)** |
| --- | --- |
| African, African American, African Caribbean, or Caribbean | 12 (4.08%) |
| Central American^2^ | 39 (13.27%) |
| Mexican^3^ | 33 |
| South American^4^ | 5 (1.70%) |
| Hispanic | 94 (31.97%) |
| Latino/a/Latin American | 19 (6.46%) |
| Middle Eastern/North African | 5 (1.70%) |
| Multiple races^5^ | 18 (6.12%) |
| Puerto Rican | 18 (6.12%) |
| Unknown, Unspecified, or Other | 84 (28.57%) |

^1^Active duty + retired military or dependent beneficiaries who did not indicate races in addition to “Other” race (e.g., participants only checked ‘Other’).

^2^Includes participants who identified as Belizean, Cuban, El Salvadorian, Mexican, or Panamanian.

^3^Also Includes participants who identified as Chicano.

^4^Includes participants who identified as Brazilian, Colombian, or Guyana.

^5^Includes participants who wrote in multiple racial or ethnic categories.
